# A novel computed method to reconstruct the bilateral digital interarticular channel of atlas and its use on the anterior upper cervical screw fixation

**DOI:** 10.7717/peerj.1737

**Published:** 2016-02-23

**Authors:** Ai-Min Wu, Wenhai Wang, Hui Xu, Zhong-Ke Lin, Xin-Dong Yang, Xiang-Yang Wang, Hua-Zi Xu, Yong-Long Chi

**Affiliations:** 1 Department of Spinal Surgery, Second Affiliated Hospital of Wenzhou Medical University, The Key Orthopaedic Laboratory of Zhejiang Province, Wenzhou, Zhejiang, China; 2 Department of Anatomy, Wenzhou Medical University, Wenzhou, China

**Keywords:** Interarticular channel of atlas, Imaging reconstruct, Anterior transarticular screws, Anterior occiput-to-axis screws

## Abstract

**Purpose.** To investigate a novel computed method to reconstruct the bilateral digital interarticular channel of atlas and its potential use on the anterior upper cervical screw fixation.

**Methods.** We have used the reverse engineering software (image-processing software and computer-aided design software) to create the approximate and optimal digital interarticular channel of atlas for 60 participants. Angles of channels, diameters of inscribed circles, long and short axes of ellipses were measured and recorded, and gender-specific analysis was also performed.

**Results.** The channels provided sufficient space for one or two screws, and the parameters of channels are described. While the channels of females were smaller than that of males, no significant difference of angles between males and females were observed.

**Conclusion.** Our study demonstrates the radiological features of approximate digital interarticular channels, optimal digital interarticular channels of atlas, and provides the reference trajectory of anterior transarticular screws and anterior occiput-to-axis screws. Additionally, we provide a protocol that can help make a pre-operative plan for accurate placement of anterior transarticular screws and anterior occiput-to-axis screws.

## Introduction

The unique osseous anatomy of the atlantoaxial complex, as well as the risk of injury to the surrounding neurologic and vascular structures, make it difficult to conduct surgery within this region (Peng et al., 2009). Most methods of upper cervical internal fixation are performed via posterior approaches (Abumi et al., 1999; Deen et al., 2003; Dickman & Sonntag, 1998).

However, sometimes a history of posterior surgery alters the osseous anatomy and leaves a few options for achieving safe and effective posterior stabilization (Dvorak et al., 2003a). Elgafy et al. (2010) reported that about 10–23% of patients requiring atlantoaxial arthrodesis had anatomic variations of vertebral artery on at least one side and were not suitable for posterior transarticular screw; the rate was about 40% in the study by Lau et al. (2010).

Anterior transarticular screw fixations (Sen et al., 2005) and anterior occiput-to-axis screw fixations were reported (Dvorak et al., 2003a) as the salvage methods. However, the osseous anatomy of the interarticular area between the superior articular fovea and the inferior articular facet of atlas was irregular; the optimal bilateral interarticular channel could not be studied on CT reconstructive images (Only the surface diameters, height and length of the targeted bone could be measured on CT reconstructive images). Therefore, we try to use two reverse engineering software packages (image-processing software and computer-aided design software) to help us to extract the approximate and optimal digital interarticular channel of atlas.

## Materials and Methods

This research was performed following the principles described in the Declaration of Helsinki and is approved by the Institutional Ethics Review Board of our hospital (No. 2013-17). Written informed consent was obtained from all participants. Two reverse engineering software packages: Image-processing software—Mimics v10.01 (Materialise, Leuven, Belgium) and computer-aided design (CAD) software—UG Imageware v13.2 (EDS, Plano, Texas, USA) were used in this study.

We studied the CT (Computed tomography) scans of 60 participants (30 Chinese males and 30 Chinese females) who were diagnosed with oral disease and with normal upper cervical spines, the CT images were obtained using Philips Brilliance 16 (Philips Medical Systems, Eindhoven, the Netherlands) with thickness of 0.45 mm.

Patients with any spinal abnormalities such as fractures, dislocations or tumors were excluded. Their scans in DICOM format were imported into image-processing software for three-dimensional (3D) reconstruction, the thresholding was set as “226-Max.” The osseous constructions of C0–C2 (Occipital-to-Axis) were separated from the skull and then exported and saved in Binary STL format.

**Figure 1:**
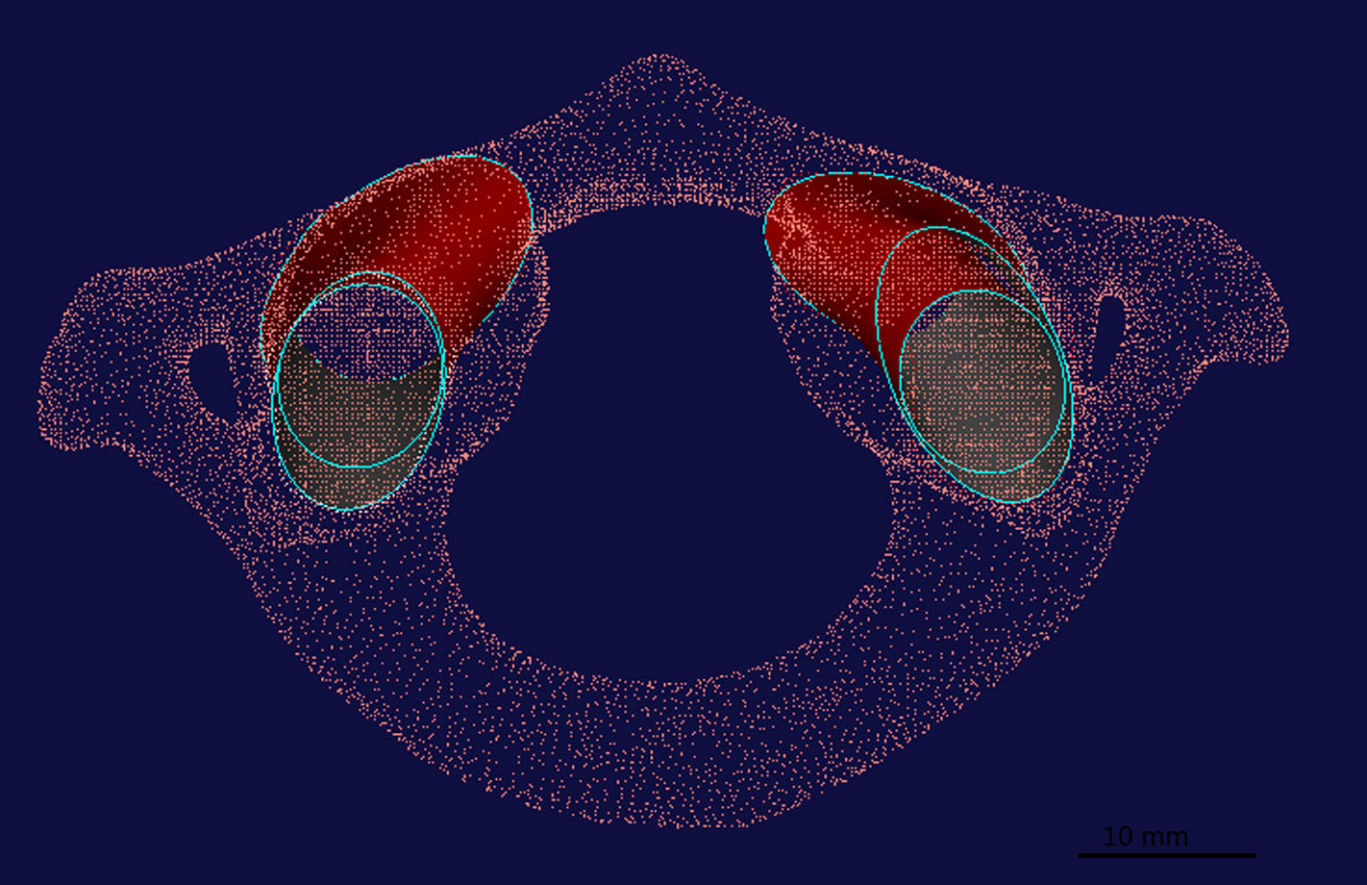
In the UG Imageware software v13.2, one hundred points (The red points) are extracted from the border line of articular facet between C0–C1, and C1–C2. Then, ellipses and inscribed circles (The blue lines) from every borderline point cloud of articular facet. The ellipses and inscribed circles below and above the C1 are used to create “loft curved surface” (red surface) both right and left sides, respectively.

**Figure 2:**
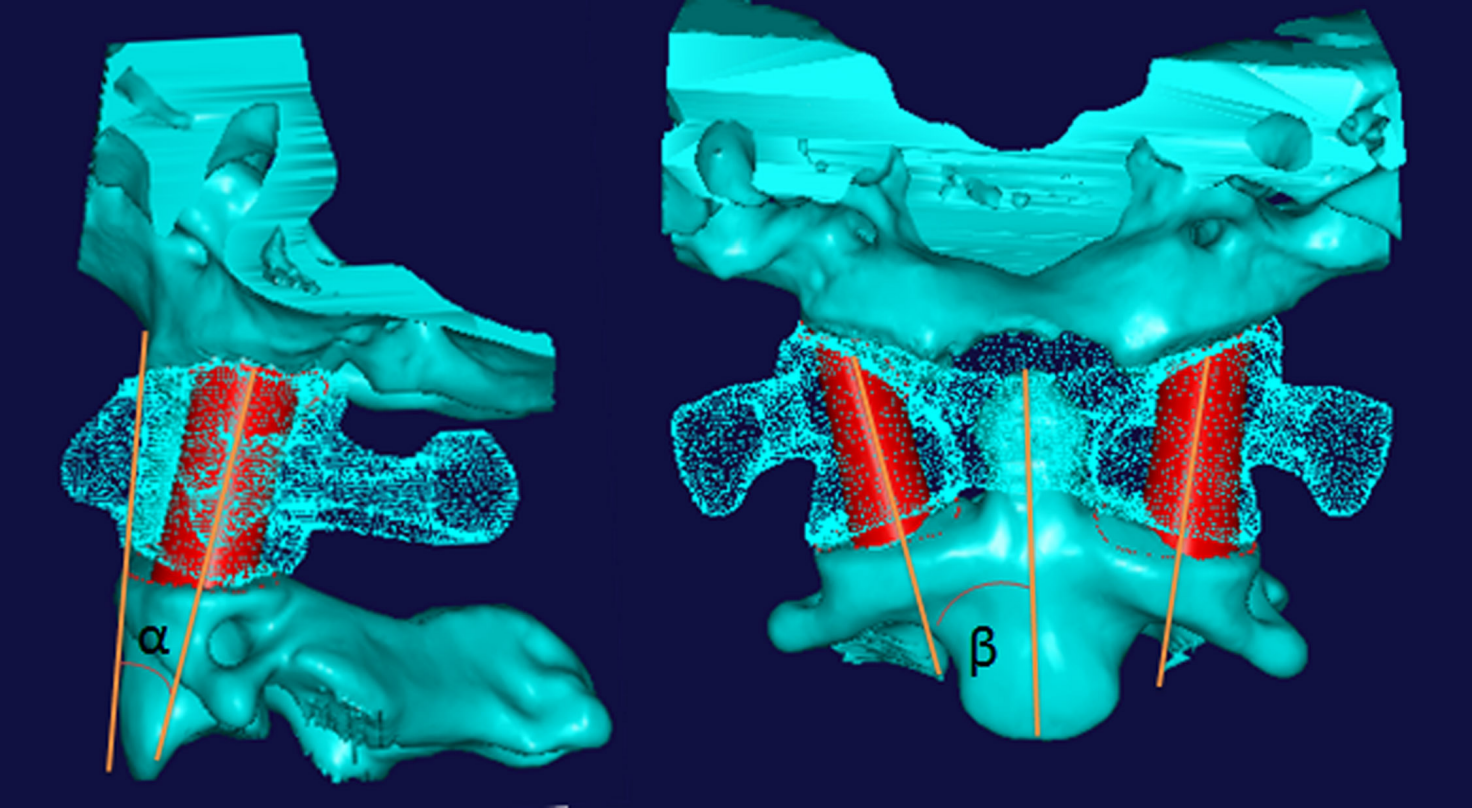
The optimal digital interarticular channel shown on lateral view and anteroposterior view. The anterior borderline between the C0 and C2 is used as reference line on lateral view, the angle between axis of loft curved surface and reference line on lateral view is measured and named as angle “*α*.” The center line of dens is used as reference line on anteroposterior view, the angle between axis of loft curved surface and reference line on anteroposterior view was measured and named as angle “*β*.”

**Figure 3:**
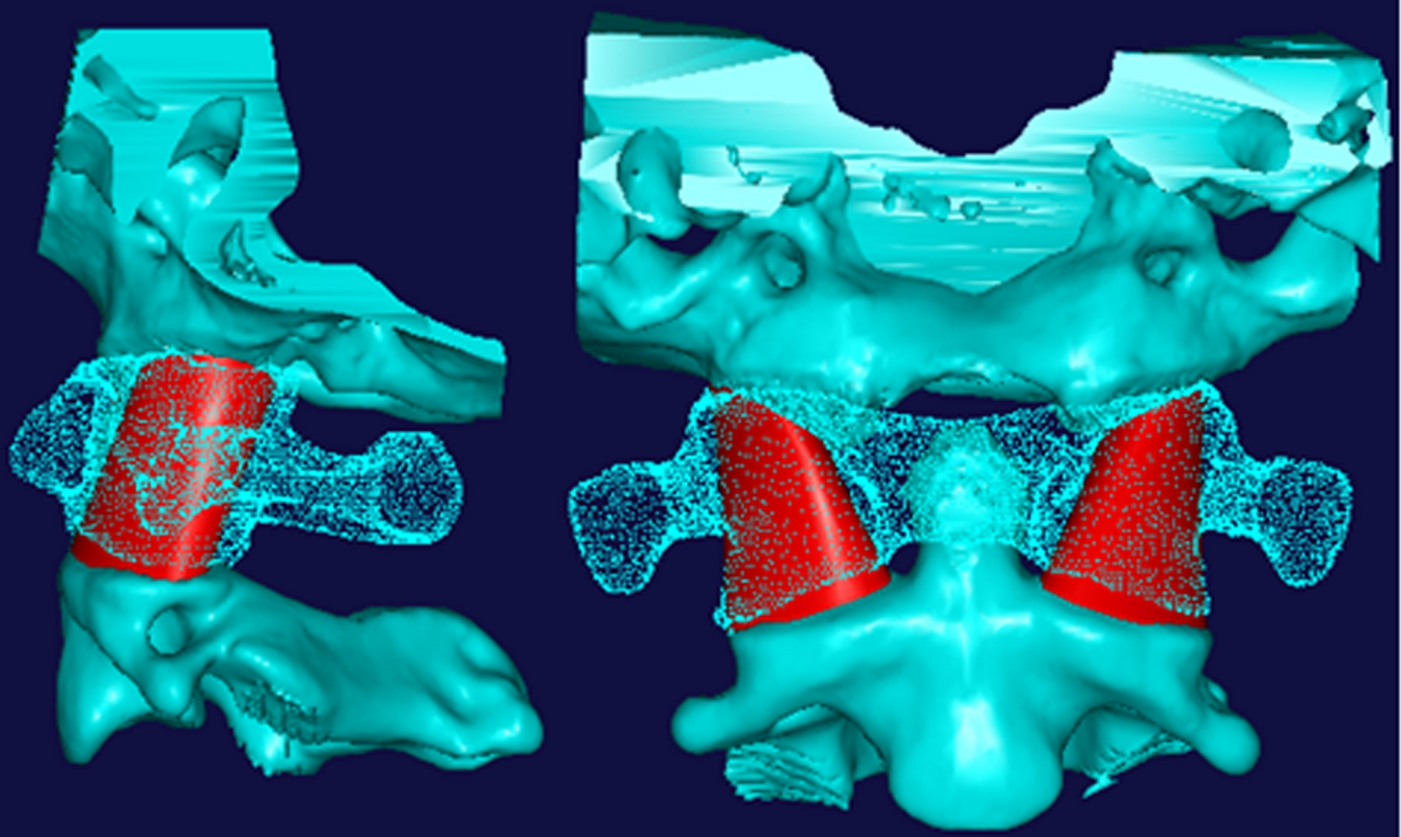
The approximate digital interarticular channel shown on lateral view and anteroposterior view.

The .STL format data was imported into CAD software. At articular facet of C0-1, one hundred points were extracted from the borderline of upper facet of C1; at the articular facet of C1-2, one hundred points were extracted from the borderline of upper facet of C2, the points at the borderline of the upper facet of C1 were manually selected by experienced surgeons. The function “Construct-Curve from cloud” (algorithm of minimum product method) was used to create ellipses and inscribed circles from every borderline point cloud of the articular facet (Fig. 1). Then, the ellipses below and above the C1 were used to create “loft curved surface” on both right and left sides, as well as inscribed circles (Fig. 1). We defined the loft curved surface from the inscribed circle as the optimal digital interarticular channel (Fig. 2), and the loft curved surface created from ellipses as the approximate digital interarticular channel (Fig. 3).

The center line of dens was used as a reference line on the anterioposterior view, and the anterior borderline between the C0 and C2 was used as the reference line on the lateral view. The angle between the axis of the loft curved surface and the reference line on lateral view was measured and named as angle “*α*” and the angle between the axis of the loft curved surface and reference line on anterioposterior view was measured and named as angle “*β*” (Fig. 2), both angle “*α*” and “*β*” were measured by screen ruler . At the same time, the diameters of the circles, the long axis and the short axis of the ellipses were also measured on CAD software.

### Statistical analysis

The data were imported into the SPSS software (version 17.0, SPSS Inc., Chicago, IL, USA) for analysis. Comparison of the data between the left and right was using paired *t*-test, comparison of the data between male and female was using two samples *t*-test. The level of significance was set at *P* < 0.05. The normalcy of data was tested by One-Sample Kolmogorov–Smirnov Test. The results were represented as “Mean ± Standard Deviation.”

## Results

All the data was normally distributed, all of *P* values of One-Sample Kolmogorov–Smirnov Test result were >0.05 (Data S1).

### Approximate digital interarticular channel

Of total 60 samples the mean *α* and *β* angles of left side were 14.38 ± 2.66° and 15.29 ± 2.91°; of right side were 14.63 ± 2.48° and 15.34 ± 2.96°. The mean lengths of long axis of C0-1, short axis of C0-1, long axis of C1-2, and short axis of C1-2 of left side were 15.89 ± 3.02 mm, 12.14 ± 2.71 mm, 17.22 ± 2.71 mm, 13.28 ± 2.75 mm, respectively; of right side were 16.33 ± 2.55 mm, 12.33 ± 2.42 mm, 16.87 ± 2.58 mm, and 13.18 ± 2.45 mm, respectively. When comparing the left side and right side of all above six parameters, no statistically significant differences were observed.

The mean *α* angles were 14.82 ± 2.37° (left), 15.05 ± 2.19° (right) in the male sample and 13.95 ± 2.89° (left), 14.22 ± 2.71° (right) in the female sample. No significant difference was found by gender-specific analysis (Table 1).

**Table 1:** The angles and axes of approximate digital interarticular channel and fitted ellipses, and results of gender-specific analysis. (Male: *N* = 30; Female: *N* = 30). *P* value is male/female *t* test.

		*α*	*β*	Long axis of C0-1	Short axis of C0-1	Long axis of C1-2	Short axis of C1-2
Left	Male	14.82 ± 2.37	15.25 ± 3.07	17.30 ± 3.11	13.36 ± 2.79	18.78 ± 2.26	14.75 ± 2.39
	Female	13.95 ± 2.89	15.33 ± 2.78	14.47 ± 2.17	10.92 ± 2.01	15.66 ± 2.19	11.82 ± 2.29
	*P* value	0.207	0.914	0.000	0.000	0.000	0.000
Right	Male	15.05 ± 2.19	15.49 ± 3.02	17.49 ± 2.20	13.43 ± 2.22	18.14 ± 2.26	14.20 ± 2.25
	Female	14.22 ± 2.71	15.20 ± 2.95	15.18 ± 2.38	11.22 ± 2.11	15.60 ± 2.25	12.15 ± 2.22
	*P* value	0.199	0.707	0.000	0.000	0.000	0.001

The mean *β* angles were 15.25 ± 3.07°(left), 15.49 ± 3.02°(right) in the male sample and 15.33 ± 2.78°(left), and 15.20 ± 2.95°(right) in the female sample. No significant difference was found in the gender-specific analysis (Table 1).

The mean lengths of long axis of C0-1 were 17.30 ± 3.11 mm (left), 17.49 ± 2.20 mm (right) in the male sample and 14.47 ± 2.17 mm (left), 15.18 ± 2.38 mm (right) in the female sample; and the mean lengths of the short axis of C0-1 were 13.36 ± 2.79 mm (left), 13.43 ± 2.22 mm (right) in the male sample and 10.92 ± 2.01 mm (left), 11.22 ± 2.11 mm (right) in the female sample. The gender-specific analysis revealed a longer measurement of both long and short axes of C0-1 ellipses in males (Table 1).

The mean lengths of long axis of C1-2 were 18.78 ± 2.26 mm (left), 18.14 ± 2.26 mm (right) in the male sample and 15.66 ± 2.19 mm (left), 15.60 ± 2.25 mm (right) in the female sample; and the mean lengths of the short axis of C1-2 were 14.75 ± 2.39 mm (left), 14.20 ± 2.25 mm (right) in the male sample and 11.82 ± 2.29 mm (left), 12.15 ± 2.22 mm (right) in the female sample. The gender-specific analysis revealed a longer measurement of both long and short axes of C1-2 ellipses in males (Table 1).

### Optimal digital interarticular channel

Of total 60 samples the mean *α* and *β* angles of left side were 12.46 ± 2.40°and 14.91 ± 3.15°; of right side were 12.87 ± 2.39°and 15.04 ± 2.96°. The mean lengths of diameter of C0-1, C1-2 were 11.55 ± 2.76 mm and 12.68 ± 2.70 mm; of right side were 11.79 ± 2.51 mm and 12.57 ± 2.60 mm. When comparing the left side and right side of all above four parameters, no statistically significant differences were observed.

The mean *α* angles were 12.60 ± 2.67° (left), 13.21 ± 2.45°(right) in the male sample and 12.31 ± 2.14°(left), 12.54 ± 2.32°(right) in the female sample; while the mean *β* angles were 14.95 ± 3.33°(left), 15.36 ± 3.14° (right) in the male sample and 14.86 ± 3.02°(left), 14.71 ± 2.79°(right) in the female sample. No significant difference was found in both *α* and *β* angles by gender-specific analysis (Table 2).

**Table 2:** The angles and diameters of optimal digital interarticular channel and fitted inscribed circles, and results of gender-specific analysis. (Male: *N* = 30; Female: *N* = 30). *P* value is male/female *t* test.

		*α*	*β*	Diameters of C0-1	Diameters of C1-2
Left	Male	12.60 ± 2.67	14.95 ± 3.33	12.65 ± 2.83	13.97 ± 2.48
	Female	12.31 ± 2.14	14.86 ± 3.02	10.45 ± 2.23	11.39 ± 2.31
	*P* value	0.644	0.908	0.001	0.000
Right	Male	13.21 ± 2.45	15.36 ± 3.14	12.78 ± 2.49	13.76 ± 2.40
	Female	12.54 ± 2.32	14.71 ± 2.79	10.81 ± 2.13	11.37 ± 2.45
	*P* value	0.279	0.401	0.002	0.000

The diameters of C0-1 were 12.65 ± 2.83 mm (left), 12.78 ± 2.49 mm (right) in the male sample and 10.45 ± 2.23 mm (left), 10.81 ± 2.13 mm (right) in the female sample; while diameters of C1-2 were 13.97 ± 2.48 mm (left), 13.76 ± 2.40 mm (right) in the male sample and 11.39 ± 2.31 mm (left), 11.37 ± 2.45 mm (right) in the female sample. The gender-specific analysis revealed longer diameters of both C0-1 and C1-2 inscribed circles in males (Table 2).

## Discussion

The complex anatomy of upper cervical spine requires accurate internal screw trajectory at this region, we use the reverse engineering software to reconstruct the digital interarticular channel of atlas for purpose to provide the anatomic information of upper cervical spine and improve the accurate placement of anterior transarticular/occiput-to-axis screw fixation.

With the help of image-processing software and computer-aided design software, we create both ellipses and inscribed circles. The loft curved surface based on ellipses give an approximate digital screw channel, and therefore, we name it as the “approximate digital interarticular channel.” The loft curved surface based on inscribed circles, we name it as the “optimal digital interarticular channel”; the later one is narrower, but more accurate.

Compare the left side and right side of both axes and angles of “approximate digital interarticular channel” and diameters and angles of “optimal digital interarticular channel,” we find that they are left–right symmetry. To compare the difference between males and females, we find that no difference is observed in angles of both “approximate digital interarticular channel” and “optimal digital interarticular channel. However, both the long axis and short axis of “approximate digital interarticular channel” and diameters of “optimal digital interarticular channel” in males are longer than those in females. The diameters of screws used for upper cervical fixation are about 3.5 mm and 4.0 mm. Therefore, there is enough space to insert the anterior transarticular screws and anterior occiput-to-axis screws when one screw is used for one side. If the positions of screws are arranged reasonably, one side with two screws is also acceptable, and we could consider choosing a larger diameter screw for males.

Anterior transarticular screw fixation is an alternative stabilizing atlantoaxial joint and has less potential risk of iatrogenic vertebral artery injury than posterior transarticular screw fixation (Xu et al., 2012). Compared to posterior techniques, it also has advantages of less surgical trauma, less blood loss, and shortened hospital stay (Li et al., 2010; Wang et al., 2012; Wu et al., 2013). Lu et al. (1998) provide the anatomic data of this technique measured from 30 dried human cervical spines, they suggest that the posterior angle on sagittal section is about 12.8–22.6°, and lateral angle on coronal section is 4.8–25.3°, but they use the subjective screw entry point, in our this study, we create digital interarticular channel, which is more objective, and provide two different approximate and optimal screw trajectories for surgeons. The anterior transarticular screw fixation is also supported by biomechanical studies (Lapsiwala et al., 2006; Sen et al., 2005), which prove it has the comparable biomechanical stability when compared to posterior transarticular screw fixation.

Dvorak et al. (2003a) report that they performed anterior occiput-to-axis screw fixation on a patient who has a history of posterior surgery that left significant posterior scar tissue, disrupted osseous anatomy, and few landmarks for achieving safe and effective posterior stabilization. They also do the anatomical research about this technique, but they only give us the evaluated posterior angle of 15–36°on sagittal plane and lateral angle of 10–20°on coronal plane, our interarticular channel can provide more accurate screw trajectory, making this technique safer. At the same time, they prove that anterior occiput-to-axis screw fixation could achieve comparable biomechanical stability to posterior plate with transarticular screws (Dvorak et al., 2003b). Wu et al. (2013) report that anterior occiput-to-axis screw fixation can also be performed via percutaneous approaches.

In both the techniques of anterior transarticular screw fixation and anterior occiput-to-axis screw fixation, the screws should be introduced towards to the bilateral interarticular part of atlas. In the three dimensional reconstruction images, the parameters of height and length of osseous anatomy could easily be obtained (Puchwein et al., 2013). However, the facets of both C0-1 and C1-2 are irregular, and we do not know where the optimal center points of these facets are or what the optimal screw trajectory channel is.

CAD software could firstly extract the points cloud from the borderline of articular facet, then create the best fitted inscribed circles and ellipses based on the extracted points cloud. These functions make the previously irregular facets fitted regular circles and ellipses. The function of the loft curved surface could create the digital screw channel to solve our above research problem. Moreover, it is proved that the Mimics and UG Imageware can provide accurate digital model when converting the data from CT to 3D images in Mimics (Wu et al., 2015) and cloud point in UG Imageware (Lu et al., 2009). Just as in our study, the cloud points were extracted manually; in fact, the software can’t extract the cloud point automatically, because different studies have different purposes, and the researcher who selects the cloud point should be familiar with his purpose and the screw trajectory.

In this study, we demonstrate a novel computed method to reconstruct the bilateral digital interarticular channel of atlas, and studied the radiological features of approximate digital interarticular channel and the optimal digital interarticualr channel of atlas. We also provide the reference trajectory of anterior transarticular screws and anterior occiput-to-axis screws. Additionally, we provide a protocol that can help in making a pre-operative plan for the accurate placement of anterior transarticular screws and anterior occiput-to-axis screws, and making these anterior approach techniques safer.

## Supplemental Information

10.7717/peerj.1737/supp-1 Data S1 Raw data

## Additional Information and Declarations

### Data Availability

The following information was supplied regarding data availability: The raw data was submitted as Data S1.

## References

[ref-1] Abumi K, Takada T, Shono Y, Kaneda K, Fujiya M (1999). Posterior occipitocervical reconstruction using cervical pedicle screws and plate-rod systems. Spine.

[ref-2] Deen HG, Birch BD, Wharen RE, Reimer R (2003). Lateral mass screw-rod fixation of the cervical spine: a prospective clinical series with 1-year follow-up. Spine Journal.

[ref-3] Dickman CA, Sonntag VK (1998). Posterior C1-C2 transarticular screw fixation for atlantoaxial arthrodesis. Neurosurgery.

[ref-4] Dvorak MF, Fisher C, Boyd M, Johnson M, Greenhow R, Oxland TR (2003a). Anterior occiput-to-axis screw fixation: part I: a case report, description of a new technique, and anatomical feasibility analysis. Spine.

[ref-5] Dvorak MF, Sekeramayi F, Zhu Q, Hoekema J, Fisher C, Boyd M, Goertzen DJ, Oxland TR (2003b). Anterior occiput to axis screw fixation: part II: a biomechanical comparison with posterior fixation techniques. Spine.

[ref-6] Elgafy H, Potluri T, Goel VK, Foster S, Faizan A, Kulkarni N (2010). Biomechanical analysis comparing three C1-C2 transarticular screw salvaging fixation techniques. Spine.

[ref-7] Lapsiwala SB, Anderson PA, Oza A, Resnick DK (2006). Biomechanical comparison of four C1 to C2 rigid fixative techniques: anterior transarticular, posterior transarticular, C1 to C2 pedicle, and C1 to C2 intralaminar screws. Neurosurgery.

[ref-8] Lau SW, Sun LK, Lai R, Luk MS, Ng YS, Wong NM, Lau PY (2010). Study of the anatomical variations of vertebral artery in C2 vertebra with magnetic resonance imaging and its application in the C1-C2 transarticular screw fixation. Spine.

[ref-9] Li WL, Chi YL, Xu HZ, Wang XY, Lin Y, Huang QS, Mao FM (2010). Percutaneous anterior transarticular screw fixation for atlantoaxial instability: a case series. Journal of Bone and Joint Surgery. British Volume.

[ref-10] Lu J, Ebraheim NA, Yang H, Heck BE, Yeasting RA (1998). Anatomic considerations of anterior transarticular screw fixation for atlantoaxial instability. Spine.

[ref-11] Lu S, Xu YQ, Lu WW, Ni GX, Li YB, Shi JH, Li DP, Chen GP, Chen YB, Zhang YZ (2009). A novel patient-specific navigational template for cervical pedicle screw placement. Spine.

[ref-12] Peng CW, Chou BT, Bendo JA, Spivak JM (2009). Vertebral artery injury in cervical spine surgery: anatomical considerations, management, and preventive measures. Spine Journal.

[ref-13] Puchwein P, Jester B, Freytag B, Tanzer K, Maizen C, Gumpert R, Pichler W (2013). The three-dimensional morphometry of the odontoid peg and its impact on ventral screw osteosynthesis. The Bone and Joint Journal.

[ref-14] Sen MK, Steffen T, Beckman L, Tsantrizos A, Reindl R, Aebi M (2005). Atlantoaxial fusion using anterior transarticular screw fixation of C1-C2: technical innovation and biomechanical study. European Spine Journal.

[ref-15] Wang J, Zhou Y, Zhang Z, Li C, Zheng W, Zhang Y (2012). Minimally invasive anterior transarticular screw fixation and microendoscopic bone graft for atlantoaxial instability. European Spine Journal.

[ref-16] Wu AM, Chi YL, Weng W, Xu HZ, Wang XY, Ni WF (2013). Percutaneous anterior occiput-to-axis screw fixation: technique aspects and case series. Spine Journal.

[ref-17] Wu AM, Shao ZX, Wang JS, Yang XD, Weng WQ, Wang XY, Xu HZ, Chi YL, Lin ZK (2015). The accuracy of a method for printing three-dimensional spinal models. PLoS ONE.

[ref-18] Xu H, Chi YL, Wang XY, Dou HC, Wang S, Huang YX, Xu HZ (2012). Comparison of the anatomic risk for vertebral artery injury associated with percutaneous atlantoaxial anterior and posterior transarticular screws. Spine Journal.

